# New therapeutic approaches for treatment of tularaemia: a review

**DOI:** 10.3389/fcimb.2014.00040

**Published:** 2014-03-28

**Authors:** Sandrine Boisset, Yvan Caspar, Vivien Sutera, Max Maurin

**Affiliations:** ^1^Laboratoire de Bactériologie, Institut de Biologie et de Pathologie, CHU de GrenobleGrenoble, France; ^2^Université Joseph Fourier-Grenoble 1Grenoble, France; ^3^Laboratoire Adaptation et Pathogénie des Micro-Organismes, CNRS/UJF, UMR 5163Grenoble, France

**Keywords:** tularaemia, treatment, antibiotics, immunotherapy, antibodies

## Abstract

Antibiotic treatment of tularaemia is based on a few drugs, including the fluoroquinolones (e.g., ciprofloxacin), the tetracyclines (e.g., doxycycline), and the aminoglycosides (streptomycin and gentamicin). Because no effective and safe vaccine is currently available, tularaemia prophylaxis following proven exposure to *F. tularensis* also relies on administration of antibiotics. A number of reasons make it necessary to search for new therapeutic alternatives: the potential toxicity of first-line drugs, especially in children and pregnant women; a high rate of treatment relapses and failures, especially for severe and/or suppurated forms of the disease; and the possible use of antibiotic-resistant strains in the context of a biological threat. This review presents novel therapeutic approaches that have been explored in recent years to improve tularaemia patients' management and prognosis. These new strategies have been evaluated *in vitro*, in axenic media and cell culture systems and/or in animal models. First, the activities of newly available antibiotic compounds were evaluated against *F. tularensis*, including tigecycline (a glycylcycline), ketolides (telithromycin and cethromycin), and fluoroquinolones (moxifloxacin, gatifloxacin, trovafloxacin and grepafloxacin). The liposome delivery of some antibiotics was evaluated. The effect of antimicrobial peptides against *F. tularensis* was also considered. Other drugs were evaluated for their ability to suppress the intracellular multiplication of *F. tularensis*. The effects of the modulation of the innate immune response (especially via TLR receptors) on the course of *F. tularensis* infection was characterized. Another approach was the administration of specific antibodies to induce passive resistance to *F. tularensis* infection. All of these studies highlight the need to develop new therapeutic strategies to improve the management of patients with tularaemia. Many possibilities exist, some unexplored. Moreover, it is likely that new therapeutic alternatives that are effective against this intracellular pathogen could be, at least partially, extrapolated to other human pathogens.

## Introduction

*Francisella tularensis*, the agent of tularaemia, is a facultative intracellular pathogen of humans and hundreds of animal species (including mammals, insects, arthropods, and protozoa). It is highly infectious, with only 10 bacteria able to cause a lethal infection. Two *F. tularensis* subspecies are virulent for humans: subsp. *tularensis* (type A), found mainly in North America, and subsp. *holarctica* (type B), found throughout the northern hemisphere. Both subspecies are classified as category A agents of biological threat by the Centers for Disease Control (CDC, Atlanta, Georgia, USA).

Tularaemia is considered a re-emerging disease, and recent outbreaks have been reported worldwide (Chitadze et al., 2009; Hauri et al., 2010). The mean incubation period of tularaemia is only 3–5 days (Tarnvik and Chu, 2007) and even less in case of aerosol contamination. Most clinical cases involve chronic lymphadenopathy associated with a skin, mucosa or conjunctival inoculation lesion. Less frequently, patients suffer from systemic diseases, and inhalation of infected aerosols may lead to severe pneumonia with mortality rates up to 60% (Gill and Cunha, 1997). This latter clinical form is the most feared in the context of bioterrorism. No vaccine is currently available and only a few antibiotic classes are effective to treat tularaemia patients, including the fluoroquinolones, the tetracyclines and the aminoglycosides (only streptomycin and gentamicin) (KuoLee and Chen, 2007; Hepburn and Simpson, 2008). Although no acquired resistance to these antibiotics has been described in natural strains of *F. tularensis*, many therapeutic failures and relapses have been reported (Perez-Castrillon et al., 2001; Kosker et al., 2013). Even with antibiotic treatment, tularaemia may be associated with 2% mortality (Evans et al., 1985). It is therefore essential to develop novel and effective preventive and curative treatments.

We will review the current therapeutic approaches and new therapeutic strategies (Table 1) published in the English literature.

**Table 1:** **New therapeutic alternatives evaluated against *F. tularensis* infection**.

**Drug**	**Mechanism of action/target**	**Activity on *F. tularensis***	**References**
**NEW ANTIBIOTICS**			
Ketolides	Inhibit protein synthesis by interacting with the peptidyl-transferase site of the bacterial 50S ribosomal subunit.	Effective *in vitro* against French isolates of *F. tularensis* subsp. *holarctica*	Gestin et al., 2010
Tigecycline	Inhibits protein translation by binding to the 30S ribosomal subunit and blocking entry of amino-acyl tRNA molecules into the A site of the ribosome	Effective *in vitro* against Hungarian *F. tularensis* subsp. *holarctica*	Kreizinger et al., 2013b
Grepafloxacin	Inhibit DNA synthesis by targeting DNA gyrase and topoisomerases	Highly active *in vitro* against American and	Ikaheimo et al., 2000
Trovafloxacin		Austrian *F. tularensis* subsp. *tularensis* and subsp. *holarctica* strains	Johansson et al., 2002
Sparfloxacin		Gatifloxacin effective *in vivo* in a BALB/c mouse model of *F. tularensis* Schu S4 infection	Tomaso et al., 2005
Gatifloxacin			Piercy et al., 2005; Steward et al., 2006
Linezolide	Inhibits the initiation process of protein synthesis	Effective *in vitro* against Turkish strains, Not effective against *F. tularensis* strains isolated in Hungary or in North America	Yesilyurt et al., 2011 Johansson et al., 2002
**ANTIMICROBIAL PEPTIDE**			
LL-37	Stimulates the innate immune response, increases in the production of IL-6, Il-12, IFN-gamma, and MCP-1	Moderately and transiently effective in a murine model of LVS-induced pneumonia	Flick-Smith et al., 2013
**OTHER MODULATOR OF INNATE IMMUNE RESPONSE**			
IL-12	Activates Th1 and NK cells and induces the production of IFN-gamma	Improves the clinical outcome and survival of animals infected with *F. novicida*, when co-administrated with gentamicin.	Pammit et al., 2004 Kirimanjeswara et al., 2008
AGP	Synthetic TLR4 agonist, increases the amount of intrapulmonary cytokines and chemokines	Reduced bacterial replication in the lungs, liver and spleen, and increased survival of animals infected with *F. novicida*	Lembo et al., 2008
CpG	TLR9 activator, indirectly activates NK cells resulting in cytokines and NO production	Better survival of mice infected with *F. tularensis* LVS, but no effect in mice with *F. tularensis* SCHU4 pneumonia	Elkins et al., 2009 Rozak et al., 2010
poly(I:C)	Synthetic double stranded RNA analog, TLR3 activator, induces an early and effective innate immune response	Better survival of mice infected with *F. tularensis* LVS or Schu S4 Reduction in replication of *F. tularensis* within human monocyte-derived macrophages	Pyles et al., 2010
Galantamine	Influences the immune response via the cholinergic anti-inflammatory pathway by up-regulating IFN-gamma production and down-regulating IL-6 production	Reduced mortality rates in mice infected with *F. tularensis* LVS	Pohanka et al., 2012
Acai PS	Enhances Th1 cell-related immunity	Increased survival of mice infected with *F. tularensis* Schu S4	Skyberg et al., 2012
**SPECIFIC ANTIBODIES**			
Monoclonal antibodies	Monoclonal antibodies against the LPS of *F. tularensis* LVS	Successfully used to treat LVS-induced pneumonia; No effect on mice infected with *F. tularensis* Schu S4	Lu et al., 2007 Kirimanjeswara et al., 2008 Savitt et al., 2009
Immune sera	Immune sera from mice infected via intra-nasal Schu S4 challenge, and then treated with levofloxacin Sera containing abundant immunoglobulin IgG2a	Protective in mice infected with *F. tularensis* Schu S4, when administered 24 h post-infection	Klimpel et al., 2008
Anti-MPF IgM and IgG antibodies	Antibodies directed against the membrane protein fraction (MFP) of *F. tularensis* Schu S4	Successfully used to treat mice infected with *F. tularensis* Schu S4 when combined with gentamicin	Sutherland et al., 2012

## Recommended antibiotics for tularaemia treatment (WHO guidelines)

According to the WHO guidelines (WHO, 2007), patients with severe tularaemia requiring hospitalization should receive parenteral administration of streptomycin or gentamicin. Both aminoglycosides display *in vitro* bactericidal activity against *F. tularensis* types A and B (Enderlin et al., 1994; Johansson et al., 2002; Kreizinger et al., 2013b). Aminoglycosides are associated with better outcomes and lower relapse rates (Enderlin et al., 1994). However, they can only be used parenterally, and they are occasionally associated with severe toxicity (especially ototoxicity and nephrotoxicity). Streptomycin is no longer available in many countries.

Fluoroquinolones are now advocated as first-line drugs in patients with clinical manifestations of mild to moderate severity (Ellis et al., 2002; Johansson et al., 2002). Ciprofloxacin may be given intravenously or by oral administration. *In vitro*, fluoroquinolones are highly active against *F. tularensis* subsp. *holarctica* (Syrjala et al., 1991; Scheel et al., 1993; Ikaheimo et al., 2000; Kreizinger et al., 2013b) and *F. tularensis* subsp t*ularensis* (Johansson et al., 2002). Most data are restricted to ciprofloxacin, but levofloxacin, with an oral bioavailability of over 99% and daily administration, is an attractive alternative. Murine models have shown that the intraperitoneal administration of levofloxacin is 100% effective in preventing tularaemia after *F. tularensis* challenge (Klimpel et al., 2008). Intravenous administration of levofloxacin was also successful in treating human tularaemia cases, without relapse (Limaye and Hooper, 1999; Aranda, 2001). The bioavailability and efficacy of levofloxacin against *F. tularensis* was also studied in a common marmoset model of inhalational tularaemia that more faithfully mimics human tularaemia pneumonia (Nelson et al., 2010). Orally administered levofloxacin was highly effective in preventing the onset of acute inhalation tularaemia. Meric et al. also reported that moxifloxacin was as successful as ciprofloxacin for treatment of oropharyngeal tularaemia (Meric et al., 2008). Recently, Sutera et al. obtained *in vitro* mutants highly resistant to fluoroquinolones in different *Francisella* species (*F. tularensis* subsp. *holarctica* LVS, *F. novicida*, and *F. philomiragia*) by propagating strains with increasing concentration of ciprofloxacin. All high-level resistant mutants shared cross-resistance to other fluoroquinolones tested (moxifloxacine and levofloxacine), while some also revealed striking levels of cross-resistance to other clinically relevant antibiotic classes (the aminoglycoside gentamicin and tetracyclines) (Sutera et al., 2014).

Tetracyclines, especially doxycycline, are considered a potential alternative. However, due to its bacteriostatic activity, treatment must be administrated for a minimum of 2 weeks, and higher relapse rates have been reported as compared to fluoroquinolones (Enderlin et al., 1994). Tetracyclines are classically contraindicated in children less than 8 years old because of the risk of teeth discoloration, and in pregnant women because of foetal bone toxicity.

Whatever drug is administered, failures and relapses are often associated with delayed and/or insufficiently long treatment. Suppurated lymph nodes often need to be removed surgically to obtain clinical cure (Penn and Kinasewitz, 1987; Celebi et al., 2006).

### Other antibiotics

Most beta-lactams have no bacteriostatic activity against *F. tularensis in vitro* (Tarnvik and Chu, 2007). Although third-generation cephalosporins (e.g., ceftriaxone) may be active *in vitro* against specific *F. tularensis* strains (Tomaso et al., 2005), therapeutic failures with these antibiotics are common (Cross and Jacobs, 1993). The ineffective activity of the beta-lactams against *F. tularensis* may be related both to the secretion of a class A beta-lactamase by this bacterium (Antunes et al., 2012) and to the poor penetration of these antibiotics in eukaryotic cells (Maurin et al., 2000). Thus, the beta-lactams are considered unreliable for tularaemia treatment.

It was first assumed that North American isolates of *F. tularensis* were naturally susceptible to macrolides, whereas most European strains were naturally resistant, including the vaccine strain *F. tularensis* LVS (Kudelina and Olsufiev, 1980). Later, Georgi et al. showed that *F. tularensis* subsp. *holarctica* isolates could be split into two biovars according to natural susceptibility or resistance to erythromycin. Strains with high-level resistance to erythromycin (i.e., biovar II) have spread to the northern and eastern parts of Europe (including Scandinavia, the Balkans, etc.), but also to Asia. The macrolide-susceptible strains (i.e., biovar I) predominate in western and southern Europe (France, Switzerland, Spain) and in North America. There is an overlap in the geographical distributions of both types of biovars, such as in Germany (Georgi et al., 2012). Nevertheless, failures and relapses in tularaemia patients treated with a macrolide have been reported worldwide, including in France (Maurin et al., 2011). Note that all isolates tested by Garcia del Blanco et al. during an outbreak in Spain were resistant to erythromycin (Garcia del Blanco et al., 2004). Thus, erythromycin is not a reliable option for treatment of tularaemia. Azithromycin is more active *in vitro* and has recently been proposed as a possible alternative in pregnant women in areas where the susceptible type B biovar I strains predominate (Dentan et al., 2013).

Although *F. tularensis* strains are susceptible to rifampicin *in vitro* (Tomaso et al., 2005), this antibiotic is not recommended for treatment of tularaemia because of a high risk of emergence of resistance during therapy. Chloramphenicol is less effective *in vitro* against *F. tularensis*, and it is associated with high relapse rates as well as bone marrow toxicity (Enderlin et al., 1994; Ikaheimo et al., 2000).

### New antibiotics

#### Ketolides

The ketolide compounds (telithromycin and cethromycin) are a subclass of macrolide antibiotics, which have been designed to address the problem of macrolide resistance in respiratory pathogens such as *Streptococcus pneumoniae*. Telithromycin was more effective *in vitro* against French isolates of *F. tularensis* subsp. *holarctica* than erythromycin (Gestin et al., 2010). Cethromycin has comparable tissue penetration, pharmacokinetics and *in vitro* activity to telithromycin, especially against bacterial species responsible for community-acquired pneumonia (Hammerschlag and Sharma, 2008). Compared with other macrolides, these compounds have different advantages, including a higher affinity for the 50S ribosomal subunit and a better stability at acidic pH (Hammerschlag and Sharma, 2008), which may partly explain the better activity in the intracellular environment of phagocytic cells where *F. tularensis* multiplies. Both telithromycin and cethromycin are potential alternatives for treatment of *F. tularensis* infection, but their clinical use in tularaemia patients has never been reported. Some authors have emphasized that cross-resistances between macrolide compounds may rapidly occur (Georgi et al., 2012).

#### Glycylcyclines

Tigecycline reaches high intracellular concentrations in tissues, macrophages and neutrophils, which makes this agent an interesting alternative for the treatment of intracellular pathogens (Pankey, 2005). Two papers report that *F. tularensis* strains isolated in Hungary and in Turkey were susceptible to tigecycline (Yesilyurt et al., 2011; Kreizinger et al., 2013a). These *in vitro* data suggest the potential usefulness of this antibiotic in tularaemia patients, but further *in vivo* experiments are needed for confirmation.

#### New fluoroquinolones

Ikäheimo et al. evaluated the *in vitro* antimicrobial susceptibility of 38 type B strains of *F. tularensis* isolated from human samples and dead wild animals. Ciprofloxacin, levofloxacin, grepafloxacin, and trovafloxacin had similar low MICs (Ikaheimo et al., 2000). Johansson et al. (Johansson et al., 2002) showed that gatifloxacin, grepafloxacin and sparfloxacin, were highly active against types A and B strains of *F. tularensis* isolated in different regions of the US. An Austrian study tested the susceptibility of 50 *F. tularensis* strains mainly isolated from hares to a wide range of antibiotics. The results showed low MIC values for all six fluoroquinolones tested (ciprofloxacin, levofloxacin, moxifloxacin, gatifloxacin, sparfloxacin) (Tomaso et al., 2005). The *in vivo* efficacy of gatifloxacin compared with ciprofloxacin and moxifloxacin was assessed in a BALB/c mouse model of *F. tularensis* Schu S4 infection. Lower mortality rates were found for gatifloxacin and moxifloxacin as compared to ciprofloxacin (Piercy et al., 2005; Steward et al., 2006).

#### Linezolid

Although linezolid is used for treatment of infections caused by Gram-positive bacteria, a Turkish study showed that *F. tularensis* could be susceptible to linezolid *in vitro* (Yesilyurt et al., 2011). However, linezolid was not effective against *F. tularensis* strains isolated in Hungary and North America (Johansson et al., 2002).

#### Liposome delivery of antibiotics

Treatment failures and relapses may also be related to the poor penetration and accumulation of antibiotics in tissues, especially in the subcellular compartment where *F. tularensis* multiplies. The liposome formulation of antibiotics is usually more effective than the conventional formulations against intracellular bacteria (Conley et al., 1997). Two studies have evaluated the liposome delivery of fluoroquinolones in order to reach high therapeutic doses of antibiotics within the intracellular compartment. One study demonstrated the efficacy of liposome-encapsulated ciprofloxacin, delivered to the lungs through aerosol inhalation, in mice with *F. tularensis* pneumonia (Conley et al., 1997). Another study showed that IV injection of liposome-encapsulated ciprofloxacin resulted in higher serum levels of this antibiotic, as well as increased drug retention in lungs, liver and spleen compared with the free drug. They also confirmed that aerosol inhalation of liposome-encapsulated ciprofloxacin, given for prophylactic or therapeutic purposes, provided mice with complete protection against a lethal *F. tularensis* pulmonary infection (Wong et al., 2003). The enhanced therapeutic efficacy can be attributable to the increased retention of ciprofloxacin in the lungs and to better intracellular delivery of ciprofloxacin by liposomes (Wong et al., 2003). The major drawback of this new therapeutic strategy is the need for more efficient techniques for preparing liposomal nanoparticles with great potential in targeting of antibiotics to bacteria and with high safety for humans (Hallaj-Nezhadi and Hassan, 2013).

### Antimicrobial peptides

The human cathelicidin antimicrobial peptide LL-37 provides protection through host immunomodulation. It selectively regulates gene activation for expression of cytokines, chemokines and their receptor to control the recruitment of leukocytes to the infection site (Scott et al., 2002). It also activates or blocks TLR signaling (Into et al., 2010) and modulates apoptosis in neutrophils and epithelial cells (Barlow et al., 2011). Flick et al. evaluated the protective effect of LL-37 against *F. tularensis* LVS infection in cell culture and murine models. The objective was to overcome the immunosuppressive effects of *F. tularensis* infection by stimulating the innate immune response. Following LVS intranasal challenge in mice, the administration of LL-37 resulted in an increased production of IL-6, Il-12, IFN-gamma, and MCP-1, and thereby reduced proliferation of bacteria in the lungs 48–72 h post-challenge. However, LL-37 was only moderately and transiently effective, and mice ultimately succumbed to infection. The immune potentiating activity of LL-37 suggests it could be used as an adjunct therapy in the treatment of persistent forms of tularaemia (Flick-Smith et al., 2013).

## Modulation of the innate immune response and suppression of *F. tularensis* replication

The pathogenicity of *F. tularensis* is correlated with its ability to evade or suppress the activation of the host immune system, and to replicate in phagocytic cells (Bosio et al., 2007). The rapid induction of the innate immune response is critical in controlling the early spread of intracellular pathogens. IFN-gamma and TNF-alpha can activate macrophages *in vitro* to restrict the replication of *F. tularensis* (Skyberg, 2013). IL-12 and Toll-like receptor (TLR) signaling are also involved in IFN-gamma and TNF-alpha production. One possible way to improve the efficacy of tularaemia treatment, and reduce the dosage and duration of the antibiotic treatment, is to combine cytokines with first-line antibiotic drugs. Indeed, different studies support the hypothesis that increasing the production of pro-inflammatory cytokines at the early stage of infection can be beneficial. This review focuses on immune agonists that, when administered after *F. tularensis* challenges, prevent or attenuate the consequences of infection and thus could be used as a curative therapeutic adjuvant in tularaemia patients. Prophylactic aspects have been presented in other reviews (KuoLee and Chen, 2007; Hepburn and Simpson, 2008; Skyberg, 2013). This approach has been used successfully by Pammit et al. (2004), who showed that although IL-12 alone was not able to reduce bacterial burdens in mice infected with *F. novicida*, intranasal IL-12 treatment decreased spleen and liver bacterial burdens more than 100-fold and lung bacterial burdens 50-fold when co-administrated with gentamicin. This combination improved clinical outcome and survival of animals when administrated early (8–24 h) after infection (Pammit et al., 2004). It should be mentioned that results obtained with *F. novicida*, a highly virulent bacterium in mice but non-virulent in humans, perhaps cannot be extrapolated to human infections caused by *F. tularensis*. Treatment of mice with IL-12 resulted in an early IFN-gamma response that allows the recruitment of neutrophils to the lungs during the first 24 h of infection. Neutrophils contribute to the rapid clearance of bacteria (Kirimanjeswara et al., 2008).

The activation of TLR signaling pathways in phagocytic cells has been reported to be a major defence mechanism against *Francisella* infection (Cowley and Elkins, 2011). Lembo et al. demonstrated that intranasal administration of a synthetic TLR4 agonist (aminoalkyl glucosaminide phosphatase, AGP) increased intrapulmonary cytokine and chemokine production. Mice treated with AGP after *F. novicida* inhalation exhibited reduced bacterial replication in the lung, liver and spleen, and increased survival (Lembo et al., 2008).

Administration of bacterial or oligonucleotide DNA containing CpG motifs to mice induced an immune activation and effective protection against a lethal parenteral challenge of these animals with *F. tularensis* LVS. This protection was highly dependent on B lymphocyte cells and INF-gamma production. Furthermore, animals surviving this lethal challenge developed a pathogen-specific secondary immunity (Elkins et al., 1999). More recently, these authors showed that DNA-related protection against LVS infection was dependent on TLR9, which is expressed intracellularly in murine macrophages, dendritic cells and B cells. Surprisingly, their data indicated that the role of B cells was not related to the action of CpG DNA motifs but to the anti-LPS antibody production by these cells in the peritoneal cavity. Instead, the production of soluble mediators by NK cells primed with CpG DNA *in vivo* contributed to DNA-mediated protection. Because murine NK cells do not express TLR9, *in vivo* activation was indirect and may have involved dendritic cells. In conclusion, this study suggested that indirect activation of NK cells by CpG DNA, via a TLR9-dependent pathway, resulted in the production of cytokines and NO, which controlled the intracellular growth of *F. tularensis* LVS in infected mice (Elkins et al., 2009). More recently, Rozak et al. conducted the same experiment with the more virulent SCHU4 strain. Their results revealed that CpG DNA failed to positively affect the course of *F. tularensis* SCHU4 pneumonic infection in mice (Rozak et al., 2010). These contradictory data emphasize the need for evaluation of both type A and type B *F. tularensis* strains in animal models.

Pyles et al. (2010) evaluated the effect of poly(I:C) (polyinosine polycytosine), a synthetic double-stranded RNA analog that stimulates TLR3, as a novel treatment for *F. tularensis* respiratory infection. TLR3 is expressed by respiratory epithelial cells and macrophages and can trigger the induction of the host innate immune response, including TNF-alpha, IFN-gamma, IL-8, and IL-6 secretion. Intranasal administration of poly(I:C) in mice induced an early and effective innate immune response by increasing the secretion of cytokines and the neutrophil influx in the lungs. This effect was transient but prolonged the survival of animals. Furthermore, poly(I:C) increased cytokine secretion by human monocyte-derived macrophages and significantly reduced intracellular replication of *F. tularensis* in these cells. Thus, Poly(I:C) may be a useful additive therapeutic agent in patients infected by *F. tularensis* aerosols.

Galantamine is an inhibitor of acetyl-cholinesterase used for treatment of Alzheimer's disease. Galantamine has also been reported to modify TNF-alpha levels through stimulation of the cholinergic anti-inflammatory pathway (Liu et al., 2010). The resolution of tularaemia infection is mediated by the production of IFN-gamma and the activation of macrophages, resulting in an increased production of nitric oxide synthase and killing of *F. tularensis* by these phagocytic cells. Pohanka et al. (2012) showed that galantamine could significantly influence the immune response via the cholinergic anti-inflammatory pathway by up-regulating IFN-gamma production and down-regulating IL-6 production. Mice treated with galantamine showed lower mortality rates when infected with *F. tularensis*.

Skyberg et al. (2012) reported that a natural polysaccharide extract isolated from Acai berry (Acai PS, derived from the berry of the palm tree *Euterpe oleracea* in South America) enhanced the clearance of *F. tularensis* LVS and Schu4 from human macrophages when co-cultured with autologous natural killer cells. Impaired replication of *F. tularensis* in human macrophages was related to increased production of IFN-gamma by NK cells. Intranasal administration of Acai PS to mice immediately, 24 or 48 h after a Schu4 aerosol challenge resulted in survival rates of 73, 60, and 33%, respectively. In both human and murine cells, Acai PS enhanced Th1 cell-mediated immunity. Acai PS is currently the most active immunotherapeutic agent reported in the literature for treatment of experimental pneumonia caused by type A *F. tularensis*.

The activation of the innate immunity may help antibiotherapy to eradicate *F. tularensis* in infected patients. However, activation of the innate immunity may lead to higher inflammatory response and injury of the lung tissue. The timing of administration of proinflammatory treatments requires careful management, since there is a danger that their use may exacerbate the symptoms of disease in infected patients (D'Elia et al., 2013). If treatment is initiated either just before or the same time as infection, then the induction of proinflammatroy cytokines by stimulatory molecules has beneficial effects on host survival as shown with CpG oligonucleotides (Elkins et al., 1999). Moreover, the mode of administration of such combination in humans remains to be determined. The small window for such treatment greatly limits application of this adjunctive therapy in clinical practice. It has been observed that the use of proinflammatory cytokines themselves such as IL-1β cause side effects, including generalized fatigue, headache, nausea, vomiting, myalgia, and arthralgia (Crown et al., 1991). By modulating rather than up-regulating the immune response via mechanisms such as cholinergic anti-inflammatory pathways (example of galantamine, Pohanka et al., 2012), the damaging cytokine storms can be prevented. This may allow a longer window for diagnosis and treatment (D'Elia et al., 2013) but these strategies require more in *vivo* investigation for further evaluation of their efficacy and tolerance.

## Administration of specific antibodies

Before the antibiotic era, patients suffering from tularaemia were successfully treated with xenogeneic immune sera (Elkins et al., 2007). The role of antibodies for protection against infections caused by intracellular pathogens has long been controversial, but recent studies have shown that they contribute to protection against *F. tularensis* infection (Kirimanjeswara et al., 2008; Skyberg, 2013). Kirimanjeswara et al. showed that passive intra-peritoneal transfer of immune serum in mice provided complete protection against intranasal challenge with lethal doses of LVS, even when administrated 24–48 h post-infection. These results indicate that serum antibodies may be used for both prophylactic and therapeutic purposes (Kirimanjeswara et al., 2007). Another study showed that monoclonal antibodies against the LPS of *F. tularensis* LVS could be successfully used to treat LVS-induced pneumonia (Lu et al., 2007). Unfortunately, these promising results could not be reproduced when using a *F. tularensis* type A strain. Anti-LVS antibodies failed to protect mice challenged with *F. tularensis* Schu S4. One explanation could be that the Schu S4 strain completely abolished the inflammatory responses that are required for efficient antibody-mediated bacterial clearance (Kirimanjeswara et al., 2008). Another study also showed relative efficacy for immunotherapy using monoclonal antibodies (MAbs). Most particularly, MAbs directed against LVS components could confer protection in mice challenged with this strain. However, MAbs were much less active in protecting mice challenged with the type A *F. tularensis* Schu S4 strain (Savitt et al., 2009). Klimpel et al. showed that mice infected via intra-nasal Schu S4 challenge and then treated with levofloxacin developed protective immunity. Interestingly, sera from these mice were protective when passively transferred to naive mice, even when administered 24 h post-infection. In these protective sera, the most abundant immunoglobulin class was IgG2a, suggesting that a Th1-type immune response was predominant (Klimpel et al., 2008). Sutherland et al. showed that the passive transfer of antibodies directed against the membrane protein fraction (MFP) of *F. tularensis* Schu S4, to mice infected with the same strain, resulted in complete protection when combined with gentamicin treatment. These sera contained high titers of anti-MPF IgM and IgG antibodies, comparable to those observed during natural infection. Post-exposure immunization with MPF antigens was an effective means of enhancing the activity of conventional antimicrobial therapy for pneumonic tularaemia (Sutherland et al., 2012). Immune sera are probably not effective enough to treat severe pulmonary tularaemia cases, but could be used in combination with antibiotics to obtain a more rapid response to treatment.

## Conclusion

The fear of a bioterrorism attack scenario after the September 11, 2001, incident has renewed the medical and scientific interest in *F. tularensis*, a class A bioterrorism agent. However, only limited progress has been made in the development of new tularaemia therapies. Due to the lack of an effective and safe vaccine, antibiotics remain the only strategies available for prophylaxis and treatment of tularaemia. New therapeutic alternatives are needed because of the potential toxicity of first-line drugs, especially in children and pregnant women; high rates of treatment relapses and failures, especially for severe and/or suppurated forms of the disease; and the possible use of antibiotic-resistant strains in the context of biological threat. Among the new therapeutic strategies, no published study present large-scale screening of new molecules for inhibitory potential of *F. tularensis* growth. There is no more data in the literature concerning the use of bacteriophage against *Francisella*. Activation of the innate immune system can enhance resistance of the host to *F. tularensis* infection and could help clearance of the bacteria and disease cure when combined with the administration of antibiotics. Promising results have been obtained in animal models, but this strategy only applies if the involved *F. tularensis* strain is still susceptible to antibiotics, which may not be the case in the context of bioterrorism. Several virulence factors have been identified such as the pathogenicity island but also genes encoding type IV pili, a type II secretion system, a type VI secretion system, iron acquisition systems (Larsson et al., 2005). Bacterial virulence factors have been increasingly regarded as attractive targets for development of novel antibacterial agents. This type of approach has for example been used in *Salmonella enterica* serovar Typhimurium and led to the identification of Cytosporone B, an inhibitor of the Type III Secretion System of *Salmonella* as novel antibacterial agent (Li et al., 2013). Finally, it should be remembered that new anti-infective strategy effective against type B strains may not apply to the more severe infections caused by type A strain.

### Conflict of interest statement

The authors declare that the research was conducted in the absence of any commercial or financial relationships that could be construed as a potential conflict of interest.
